# The Emerging Burden of Genetic Instability and Mutation in Melanoma: Role of Molecular Mechanisms

**DOI:** 10.3390/cancers14246202

**Published:** 2022-12-15

**Authors:** Rashidul Alam Mahumud, Md. Shahjalal

**Affiliations:** 1 NHMRC Clinical Trials Centre, Faculty of Medicine and Health, The University of Sydney, Camperdown, NSW 2050, Australia; 2 Department of Public Health, North South University, Dhaka 1229, Bangladesh

**Keywords:** melanoma, genetic instability, burden, molecular mechanism

## Abstract

**Simple Summary:**

Melanoma is a severe skin cancer affecting thousands of people and a growing public health concern worldwide. The potential hallmarks of melanoma are genetic instability and mutation (GIAM), which is the crucial vulnerability of cancer cells, determining their sensitivity to harmful treatments, including radiation and many chemotherapeutics. Therefore, the ability of cells to sense DNA damage and respond appropriately is an essential aspect of genome maintenance.

**Abstract:**

Melanoma is a severe skin cancer affecting thousands of people and a growing public health concern worldwide. The potential hallmarks of melanoma are genetic instability and mutation (GIAM), which are driving mechanisms for phenotypic variation and adaptation in melanoma. In metastatic melanoma, DNA repair-associated genes are frequently expressed at higher levels than in primary cancers, suggesting melanoma cells rely on genetic stability to spread distantly. The tumour microenvironment is affected by genomic instability and melanoma mutation (GIMM), which plays significant roles in developing GIMM and their contributions to the overall disease burden. The GIAM is the crucial vulnerability of cancer cells, determining their sensitivity to harmful treatments, including radiation and many chemotherapeutics. The high incidence of melanoma is typically associated with genetic modifications, and several clinical and genetic interventions have been critical in easing the burden.

## 1. Introduction

Melanoma is a deadly skin cancer that represents a global important public health concern [1,2]. In 2020, it was estimated that 325,000 new cases of melanoma were detected globally, and 57,000 people died from melanoma each year [1]. If 2020 rates remain stable, the global burden from melanoma is estimated to increase to 510,000 new cases (~50%) and 96,000 deaths by 2040, an urgent issue for the global leader in managing and controlling it [1]. In addition, it established that melanoma leads to a high health burden, including years of life lost [3,4]. Furthermore, high disability-adjusted life years [5] also impose an economic burden [6,7] on the menage of melanoma, resulting in poor survivorship.

Many hallmarks contribute to this burden, including genome instability and mutation (GIAM), which can result in high-level mortality and difficulties in continuing advanced treatment. Despite a highly correlated prognosis with the stage and extent of tumours, advanced melanoma with nodal involvement or metastases has a five-year survival rate of 78%, which drops to 15% due to the burden [8]. However, early detection is still a vital strategy to diminish melanoma mortality [9], enhance survival [10], and be cost-effective [11,12]. Approximately 50% of melanoma patients are not qualified for advanced therapies or drug treatments due to the burden of GIAM [13]. Therefore, it is essential to have a comprehensive understanding of GIAM as one of the vital hallmarks of melanoma for the early diagnosis and savings of life.

The GIAM plays a fundamental role in tumorigenesis and melanoma disease progression, determining how sensitive they are to harmful treatments, including radiation and chemotherapeutics [14]. The ability of cells to sense DNA damage and respond appropriately is an essential aspect of genome maintenance. There is a significant role for molecular mechanisms in the genetic features of melanoma, which profoundly influence tumour carcinogenesis and the therapeutic responses to eliminate the burden of GIAM. Therefore, this essay will outline an overview of the role of GIAM with molecular features, highlight some potential findings based on an advanced existing study, and open questions for further research associated with melanoma, particularly GIAM. 

## 2. Genetic Instability and Mutations in Melanoma

The GIAM is one of the potential hallmarks of melanoma [15], which play a significant role in tumour evolution, acting as the driving mechanism towards phenotypic variation and adaptation in melanoma; however, it is not the only limit for melanoma. Mutations into the genome by considering accumulation and fixation, both in the engraved or controlling sequences and in those inactive, is one of the effective approaches by which evolution is carried out. The burden of GIAM is associated with DNA damage that may occur at two different mechanistic levels. Although GIAM is generally determined as an entire gene or focal aneuploidy/segmental at the chromosomal level. However, it is also detected at the nucleotide level and is exposed by alterations in specific DNA sequences along with an identical nucleotide structure, the satellite DNA loci [16,17]. The tumour cells constitute a unique microenvironment that promotes maintaining the malignant properties of the cancer cells. Gene expression differences in reactive tumour stroma might link the tumour microenvironment (TME) and tumour cells [18]. The TME is mainly characterized by hypoxia, low pH, and nutrient deprivation compared to normal tissue microenvironments. Inflammatory cells in the TME may produce reactive oxygen species (ROS), causing genetic instability. Oxidative DNA damage might occur not only in tumour cells but also in stromal cells [18,19,20]. The TME changes caused by oxidative stress may contribute several ways to the various stages of tumour progression. The mutation of melanoma also influences this TME [20]. Furthermore, these melanoma oncogenes have advanced melanoma biology understanding.

## 3. GIMM and Immunotherapy

Despite recent groundbreaking advances in the treatment of cutaneous melanoma, it remains one of the most treatment-resistant malignancies [21]. Immunotherapies represent an alternative to molecular targeted therapies. Improving the immunotherapeutic potential in most human cancers, including melanoma, requires identifying increasingly detailed molecular features underlying the tumour’s immune responsiveness and acting as disease-associated biomarkers [22]. Genomic instability is one of the hallmarks of cancer [23]. Through this acquired instability, malignant cells accumulate non-synonymous coding changes that create novel epitopes and proteins unique to the malignant genome [24]. These proteins may serve as potential targets for the host immune system by functioning as neoantigens.

## 4. The Potential Key Players in Genetic Instability and Mutations in Melanoma

The GIMM is associated with several determinants that primarily comprise genetic alterations and the TME. For example, the genesis and progression of melanoma have been linked to genetic mutations involving several genes. Whereas the TME is affected by the GIMM [20]. Among the extensive genetic alternations in GIMM, NRAS, BRAF, KIT, MITF, NEDD9, and TP53 are six signature mutations that play significant roles in developing GIMM and their contributions to the overall disease burden [20].

### 4.1. NRAS

The NRAS is one of the first genes shown to be mutated explicitly in GIMM, which occurs in about a fifth of cutaneous melanoma and imposes a high-level aggressive clinical behaviour and the burden of GIMM has increased remarkably in recent years [25,26,27]. A growing of literature has documented that the NRAS mutation is substantially related to a higher incidence of melanoma (i.e., 15–30% of melanoma) [27], which is ten times higher than repeatedly than HRAS or KRAS [20,28,29]. NRAS mutations transforming all three genes to involve oncogenes almost continuously happen in residues 12, 13, or 51 of the protein [30,31]. Additionally, the specificity of mutations in melanoma for NRAS compared to HRAS and KRAS is noteworthy, thinking that all three isoforms proceed in GIMM and melanoma-related short-term cultures [30,31].

In brief, the reappearance and high translating promise of oncogenic NRAS mutations in GIMM exhibit the significant role of this gene and genesis maintenance [20]. Several oncogenes are associated with cancer, but the invention and adoption of NRAS required years of a low-throughput experiment. However, it persists vague whether various NRAS mutations produce distinctive biological or clinical characteristics in melanoma.

### 4.2. BRAF

**Figure 1:**
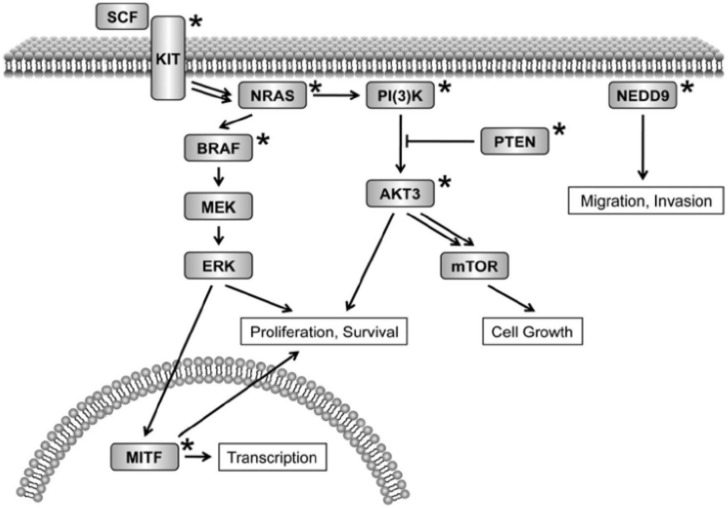
Oncogenic dysregulation in melanoma using cell signalling [20].

The BRAF is a gene that consists of RAS in terms of the MAP kinase signalling route (Figure 1), and it produces MEK to phosphorylate ERK, which enriches cell growth in melanoma. A systematic resequencing investigation involving 545 cancer cell lines revealed that oncogenic BRAF mutations are highly recurrent in melanoma [32]. It is also estimated that 59% of melanomas are associated with the BRAF mutation, significantly more than the overall incidence of mutations in cancers of 8% found in patient-originated melanoma and short-term cultures [20].

Over 90% of the BRAF mutations in melanoma consist of a single glutamic acid replacement in the kinase domain (V600E) [32]. A previous study has detected many genes against BRAF mutation in melanoma, including significant cell cycle controls, tumour permanence enzymes, and microphthalmia-related transcription components [33]. These outcomes underline the crucial function of BRAF in melanoma in terms of genesis and progression. Furthermore, the reduction in oncogenic BRAF mutation by RNA resistance in cultured melanoma cells [20], restrains proliferation and elicits apoptosis through mouse xenograft models [34,35]. Subsequently, inhibitors of BRAF mutation are of great attention as effective sighted prevention mechanisms (i.e., therapies) for melanomas sheltering BRAF mutations. As a result, numerous BRAF and MEK inhibitors are present in several stages of elucidation and clinical trials [33]. BRAF and PTEN perform in several routes against RAS, so glean mutations are strained to trigger both routes. Therefore, it investigates why BRAF would be transformed higher commonly than NRAS in melanoma.

### 4.3. MITF

The MIFT is an established and well-known melanoma oncogene, associated with a primary regulator of the melanocyte lineage and a potential biomarker for melanoma diagnosis [34,35]. This oncogene is also associated with a particular bodily alteration in this malignancy. During melanoma tumorigenesis, amplifications and deletions of essential genes that control cell growth, survival, etc., frequently function as leading causes of tumorigenesis.

Differences in DNA copy number are immediately reported at high persistence outcomes by homogenising tumour genomic DNA to microarrays consisting of oligonucleotides comprising the whole human genome [36]. A previous study found that an increased incidence of melanoma (~10–20%) among advanced tumours was contributed by the MIFT amplification that reduced five years in survival [37]. Remarkably, MITF developments are instantaneous with mutations in BRAF. The development of melanoma cell lines sheltering MITF amplification prevented subsequent RNAi-mediated of MITF or the establishment of dominant-negative MITF [37]. As a primary regulator of melanocyte growth and separation, MITF signifies a promising group of “lineage-survival” oncogenes [38]. Inconsistently, NRAS and BRAF in the context of oncogenic, which gain new and tumour-related cellular functions because of nucleotide mutations, MITF turn into oncogenic by deregulating, impacting survival systems that are also described in the general melanocyte lineage. It is well-known that natural-type MITF is necessary for lineage survival and that insufficiency of MITF findings in the lack (or failure) of melanocytes during progression [39]. Similar survival procedures regulate melanocyte proliferation, and improvement may consequently endure or be liberalised during tumour progression.

However, to understand why specific mutations occur at various incidences of different tumour types, it is indispensable to understand the role of lineage in other oncogenic changes.

### 4.4. NEDD9

The NEDD9 is the only resident gene that is upregulated when associated with untransferable, most important melanocyte cultures. It is also one of the primary pillars of integrin-related signalling cascades that initiate the FAK to stimulate cell migration and engage in effective communication across other proteins to the RAS, pointing out cascades. In addition, NEDD9 also significantly contributes to approximately one-third of the incidence of melanoma (36%) and its expression is upregulated and correlated with tumour progression [40]. A functional study shows that NEDD9 is a potential melanoma gene for invasion and metastasis [40], while another study illustrates NEDD9 outcomes regarding genetic loss in a consistent phenotype. In this context, it is noteworthy that the adverse effects of a NEDD9 genotype are primarily limited to impacts on tumorigenesis [41]. Moreover, NEDD9 is a tumour-causing factor and an aggressive biomarker that influences poor prognosis and treatment resistance in melanoma [41].

### 4.5. KIT

The KIT is an indispensable gene for melanocytes considering survival and growth [34,38]. In contrast, this gene expression generally falls for the period of melanoma progression. KIT encodes a receptor tyrosine kinase (RTK) for the stem cell factor (SCF) ligand and functions as an upstream activator of the MAP kinase signaling pathway (Figure 1). The KIT rearranging in melanomas with enlargement of 4q12 showed a significant mutations [37,41], but advanced tumour sequencing has proven that they may contribute to only 2 to 5% of cases [42,43]. The KIT mutations are most predominant in CSD (8–20%), mucosal (16–25%), and acral (12–23%) melanomas [44,45]. An earlier study documented that many additional case elaborations were deprived of identifying sequence mutations [44]. There is an immediate advantage to outlining patients for KIT mutations in melanoma patients before participating in clinical trials associated with targeted agents based on these correlations.

### 4.6. TP53 Mutations

The TP53 gene plays an essential role as the “guardian of the genome” for several reasons, including pleiotropic perform in shielding melanoma cells from genotoxic damages, DNA repair, playing as a tumour suppressor and repressor of various genes regulating cell-cycle progression, and causing programmed cell death [46,47]. TP53 mutations contribute to approximately 15% of TCGA cases, generally ultraviolet (UV). In melanoma, TP53 may have an inactivation by several approaches, including the deactivation of p14, which produces upregulation of proto-oncogene [48,49]. Furthermore, TP53 is mutated in melanomas protecting some of the most important subgroups of NRAS, BRAF or NF1 mutations. In contrast, MDM2, which desecrates p53, is heavily magnified, a possible mechanism of p53′s desecration [50]. Finally, TP53 alterations expand entirely in melanomas, indicating that these mutations may promote tumour progression substantially.

As described above, oncogenic NRAS mutations in GIMM exhibit its significance for systematic approaches to melanoma genesis and maintenance [20]. However, whether different NRAS mutations induce distinct biological or clinical features in GIMM remains unclear. While multiple initiatives have contributed to reducing the burden of melanoma, innovative treatments and genome-wide initiatives could help to reduce the burden of GIMM effectively, thus saving a thousand lives [51].

A recent study by Cai et al. (2022) suggests that PDPK1i with MEKi is an effective immunostimulatory strategy against NRAS mutant melanoma [52]. Previous genome studies [53,54] concluded that PDPK1 mRNA expression was positively correlated with NRAS mRNA expression level in GIMM patients with NRAS mutant compared to NRAS wild-type patients. It suggests that PDPK1 play a significant role in tumour-promoting performance in patients with NRAS mutant melanoma. Further, this combination of synergistic effects (PDPK1i+MEKi) demonstrated synergistic inhibition in GIMM across a wide range of drug concentrations. However, the treatment with the MEKi trametinib combined with a PDPK1i has synergistic outcomes; for instance, it has been observed that the effects of two MEK inhibitor drugs (GSK1120212 and PD0325901) have very few changes at the protein level in the tissues [55], which was consistent with trametinib, indicating broad synergistic outcomes. This data suggest that the combined effort inhibits the tumour growth of NRAS mutant melanoma synergistically. A previous study indicated that MEKi combined with immune checkpoint inhibitors might enhance survival in patients with NRAS mutant melanoma [56].

The combinative drug-resistant strategy applied a more thoughtful growth suppression and sustained survival than either initiative alone. Therefore, the development of combination therapy for treating melanoma patients is justified by targeting PDPK1 to stimulate antitumor immunity and sensitise NRAS mutant melanoma to MEK inhibition. Recently, immunotherapy, targeted therapy with cutaneous melanoma, and kinase inhibitors have enhanced survival rates for patients with cutaneous melanoma.

The burden of GIMM has been addressed through many initiatives to date. As discussed above, the combined drug-resistant in GIMM and its role in medical science includes combining current anti-melanoma drugs in GIMM with therapeutic agents. However, long-term clinical benefit is scarce due to rapidly evolving drug resistance and the dynamic nature of new medical technologies in GIMM, which may also apply to other cancers. Though it has been discovered that several genes and mediator factors are in GIMM; however, the main concern is how these findings might affect the health system in the long run to manage melanoma. It is also difficult to measure effectiveness due to adoptive and dynamic natures and the short durability of a clinical pathway. In addition, some studies stratified small groups for a particular or combined initiation, resulting in a small sample size and high-level uncertainty. However, clinical decisions may not be more productive due to the high level of uncertainty and short shelf-life of clinical initiations.

In brief, combining current anti-melanoma drugs with therapeutic agents that undermine the melanoma cell genome and inhibit DNA repair presents promise in melanoma treatment, and additional preclinical and clinical studies are necessary. In spite of the fact that it may not provide concrete solutions to these questions, these discussions are expected to stimulate further discussion and promote the research to enhance the knowledge of different therapeutic approaches for melanoma.

## 5. Conclusions

GIMM have contributed to the emerging burden of tumorigenesis and disease progression over the last decades. In addition, the GIAM is the crucial vulnerability of cancer cells, determining their sensitivity to harmful treatments, including radiation and many chemotherapeutics. Therefore, the ability of cells to sense DNA damage and respond appropriately is an essential aspect of genome maintenance. In general, the high-level incidence of melanoma was associated with genetic alternations. Several clinical and genetic initiations played a significant role in overcoming this burden. Unfortunately, this burden is emerging today due to low patient outcomes, lack of long-term effectiveness, and a combined initiation of novel therapeutic strategies. Cai and his colleagues found that PDPK1i+MEKi is an effective immunostimulatory approach counter to NRAS mutant melanoma. Genetic instability in several types of cancer contributes to acquiring a phenotype needed for colonising distant organs, and metastatic progression correlates with an increase in mutation burden and alteration of genes involved in DNA damage response [57]. We need to understand better the mechanisms that hinder a cell from tolerating genomic instability and the cellular consequences of exceeding a genomic instability limit

Future research on precise genomic and molecular sequencing is essential to incorporate a combined initiation of novel therapeutic strategies for long-term benefits targeting the abnormal GIMM to overcome the low survival and high burden of GIMM.

## Data Availability

Not applicable.
